# Stable, intense supercontinuum light generation at 1 kHz by electric field assisted femtosecond laser filamentation in air

**DOI:** 10.1038/s41377-023-01364-3

**Published:** 2024-02-02

**Authors:** Yaoxiang Liu, Fukang Yin, Tie-Jun Wang, Yuxin Leng, Ruxin Li, Zhizhan Xu, See Leang Chin

**Affiliations:** 1 State Key Laboratory of High Field Laser Physics, Shanghai Institute of Optics and Fine Mechanics and CAS Center for Excellence in Ultra-intense Laser Science, Chinese Academy of Sciences, Shanghai, China; 2 Center of Materials Science and Optoelectronics Engineering, University of Chinese Academy of Sciences, Beijing, China; 3 Centre d’Optique, Photonique et Laser (COPL) and Département de physique, de génie physique et d’optique, Université Laval, Québec, Québec, Canada

**Keywords:** Supercontinuum generation, Supercontinuum generation

## Abstract

Supercontinuum (SC) light source has advanced ultrafast laser spectroscopy in condensed matter science, biology, physics, and chemistry. Compared to the frequently used photonic crystal fibers and bulk materials, femtosecond laser filamentation in gases is damage-immune for supercontinuum generation. A bottleneck problem is the strong jitters from filament induced self-heating at kHz repetition rate level. We demonstrated stable kHz supercontinuum generation directly in air with multiple mJ level pulse energy. This was achieved by applying an external DC electric field to the air plasma filament. Beam pointing jitters of the 1 kHz air filament induced SC light were reduced by more than 2 fold. The stabilized high repetition rate laser filament offers the opportunity for stable intense SC generation and its applications in air.

## Introduction

The supercontinuum (SC) light source comes from the nonlinear propagation of ultrafast laser pulses in an optical transparent medium. It is also called “white light” laser when the SC spectrum stretches from the near ultraviolet to the infrared wavelengths. The invention and development of SC^1^ has advanced many applications, for instance, in ultrafast laser pulse generation and compression (attosecond pulse)^2^, extremely high-precision optical frequency and time metrology^3,4^, terabits/sec high-capacity information communication^5^, ultrafast laser spectroscopy^6,7^ and imaging^8^. Towards the applications, the SC light sources are mostly generated from bulk materials^1,9^ and photonic crystal fibers^10^ which limit the SC pulse energy due to the low damage threshold of solid materials. More recently gas-filled hollow-core fibers have enabled high power scaling up to ~ mJ^11,12^. SC generation in gases through ultrafast laser filamentation^13–15^ is a subject of intense studies since the first publication by Corkum et al.^16^ because it is in principle immune to damage.

Following the development of femtosecond lasers operating at high repetition rates^17^, growing attention is devoted to the high repetition rate laser filamentation^18^ because it offers new possibilities toward applications, for example, forming air waveguide^19^, penetrating fog^20^, inducing snow formation in a cloud chamber^21^, triggering discharge^18,22^ and machining materials efficiently^23^, etc. However, the milliseconds time scale of thermal diffusion in an air filament^24^ leads to air density reduction at the arrival of the next laser pulse for a kHz repetition-rate laser. The thermal self-action effect results in significant spatial and intensity jitters of the laser filament^25–27^. This constitutes a challenge for applications using kHz filament and its SC light source.

In previous works, the fluorescence signal, enhanced ionization and the lifetime of the filament under an extra electric field have been investigated^28,29^. Besides, filament guided ionic wind was observed^30^. However, the beam pointing properties of the SC light and the filament under the electric field are not explored. In this work, we solve the bottleneck problem of the self-generated jitters and report a stable, intense, kHz SC generation of a Ti-Sapphire laser through filamentation directly in air. This was accomplished by simply applying an external DC electric field on the filament’s plasma channel. Through the combined effects of suppression of plasma based thermal deposition^19^ and Coulomb interaction, both the beam pointing and the intensity jitters of a 1 kHz air filament induced SC light were reduced by more than 2-fold. This technique of reducing the thermally induced instability of high repetition rate filaments would work at other filamenting laser wavelengths due to the plasma nature inside a filament and therefore provides new opportunities for the generation of intense stable SC and its applications. Besides the SC generation, the stability improvement technique of kHz filament would be definitely useful not only to other filament based secondary sources, such as, THG^14,31^, THz^14^, air lasing^32^, but also to filament-based imaging^33^ and micro-machining of condensed materials^23,34^.

## Results

**Fig. 1:**
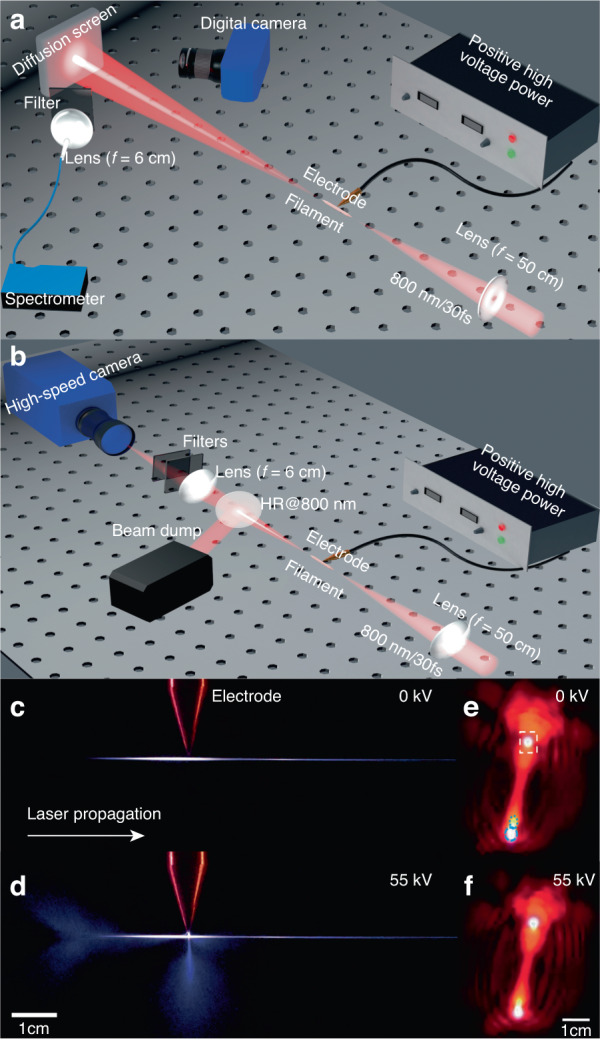
Schematic of experimental setups. **a** Generation and characterization of stable high energy SC light in air at kHz repetition rate. **b** Filament jitter measurement. Real color images of laser filament in air with high voltage off **c** and on **d**. The corresponding far-field forward beam patterns on a white screen in real color: **e** high voltage off and **f** high voltage on. Laser pulse energy was 6.12 mJ and the voltage applied on the electrode was 55 kV. The distance between the electrode tip and the laser filament was approximately 1 mm. **c**, **d** and **e**, **f** have the same length scale, respectively

A 1 kHz Ti-sapphire laser with 30 fs, 6.12 mJ per pulse was focused to generate a filament in the 30% relative humidity air by an *f* = 50 cm plano-convex lens. A copper electrode was perpendicularly applied on the filament at a distance of 1 mm from the tip of an electrode as shown in Fig. 1a, c. The DC breakdown discharge of the electrode as well as with filamentation is dependent on the relative humidity. The breakdown voltages increase when the relative humidity increases from the dry air. Multiple filaments were produced and the plasma channel was about 9 cm in length (Fig. 1c). Meanwhile, three white light spots (including the top one and bottom two) were generated in the forward direction. They are shown on the diffusion screen as marked in Fig. 1e. The three white light spots are generated by multiple filaments merged in one plasma channel which can be treated as a whole when applying the extra static electric field. The beam pointing behaviors for the three white lights merely depends on the interaction between the whole channel and the static electric field. Thus, the trend behaviors of beam pointing stability under the static electric field are coincident for the three white lights. For the sake of data processing, we chose the top white light spot (marked by the rectangle) because of its higher intensity contrast. When applying a DC voltage of 55 kV on the electrode, laser guided coronas^35,36^ were observed (Fig. 1d) by a digital camera (Nikon D7200, Fig. 1a) from the top. Far field beam patterns at approximately 1.2 m to the end of the filament were recorded by the digital camera as shown in Fig. 1f. This digital camera operating at 60 fps was also used to monitor the spatial stability of the white light laser spot on the diffusing screen. A focusing lens (2-inches in diameter, *f* = 6 cm) set at an angle of 30° to the laser propagation axis imaged the scattered light from the diffusion screen (Fig. 1a) onto a fiber coupled spectrometer (Ocean Optics HR4000) for spectral analysis. A neutral density filter was put in front of the lens to attenuate the diffused light. To monitor the filament jitter, an imaging system was constructed as shown in Fig. 1b. A plano-convex lens (*f* = 20 cm) was placed at a distance of 2-f after the filament to image it into a laser-triggered high speed camera (PCO. Dimax HS4). The high speed camera was with an exposure time of 2.5 µs. The intensity of the filamenting laser was attenuated before entering the high speed camera by a high reflectivity mirror and several neutral density filters.

### Improvement of the pointing stability of the SC light source

**Fig. 2:**
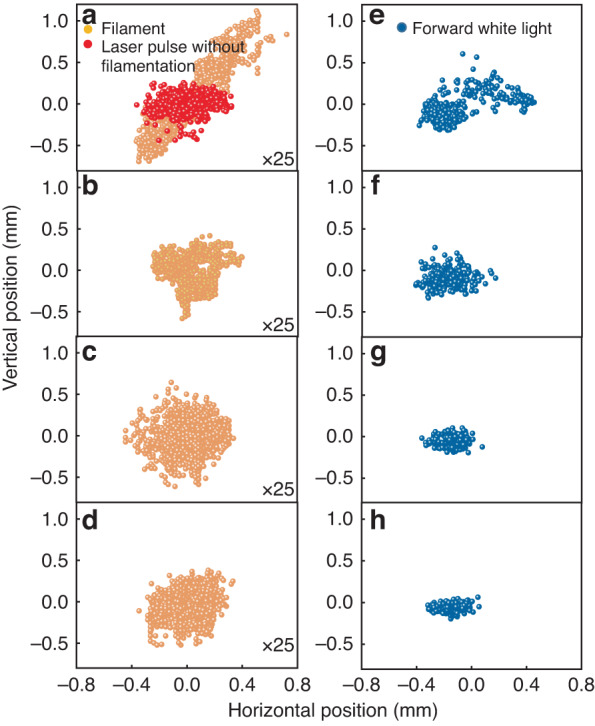
Maps of filament’s position and forward white light spots with different high voltages. **a**–**d** Filament’s position, **e**–**h** forward white light spots. **a**, **e** 0 kV; **b**, **f** 2 kV; **c**, **g** 30 kV; **d**, **h** 50 kV. The position of the optical axis without filamentation and without high voltage is defined as (0, 0)

Figure 2 shows the statistical center positions of diameter ~100  μm filament cross sections (orange dots), ~5 mm diameter white lights (blue dots) and ~2.5 cm diameter free propagated beams (red dots), respectively. Figure 2a (red dots) shows the fluctuation of the spatial positions of the initial laser beam (propagating freely without filamentation and the high voltage) which should be contributed by laser itself and air disturbance in the lab. The fluctuation of the spatial positions of the filament at the focal zone without the electric field (orange dots in Fig. 2a) is much larger than the initial laser beam. Figure 2e shows the white light laser spots on the scattering screen as measured in Fig. 1a without the electric field. It is obvious that the beam pointing of the forward white light laser beams appear in a larger area (Fig. 2e) due to the wandering nature of the kHz repetition rate filaments^25–27^.

Once the electric field was applied near the filament, the pointing scattering of the filament and that of the white light laser became smaller (Fig. 2b–d, Fig. 2f–h) indicating the improvement of pointing stability as the voltage was tuned from 1 kV up to 50 kV. It is noted that Fig. 2a–d are magnified by 25 times as for a comparison with Fig. 2e–h in the same scale. The time averaged current measured at the electric field producing electrodes is large enough to cause the DC current to sag (see the results provided in the Methods).

**Fig. 3:**
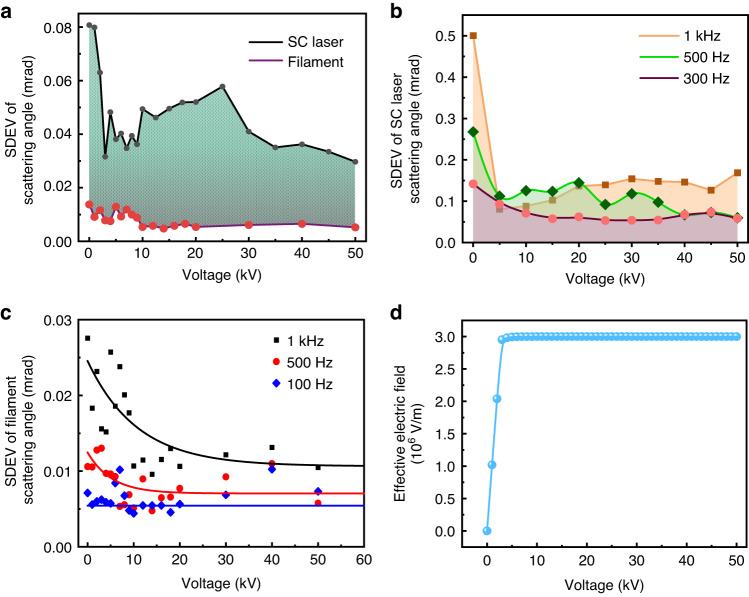
Pointing stability of the forward SC laser and filament as a function of the applied high voltage. **a** SDEVs of the forward SC laser and filament pointing angles at 1 kHz. SDEVs of the scattering angles of forward SC light (**b**) and filament (**c**) under different laser repetition rates. Solid lines in (**c**) are fits for guiding the eyes. **d** Simulated result of the effective electric field applied on the filament as the voltage increasing

The standard deviations (SDEVs) of the scattering angles of the white light lasers and those of the filaments are shown in Fig. 3a. The dithering of the forward white light was larger than that of the filaments. The jittering of the filament and the white light are due to the thermal self-heating coming from the recombination of ions and electrons inside the filament. The pointing stability of the white light was improved by approximately 2 times as the voltage increased from 0 to about 4 kV and tended to be stable beyond 4 kV (Fig. 3a). This is because the effective electric field applied on the filament increases sharply as the applied voltage increases (as shown in Fig. 3d) and it stabilizes beyond about 4 kV (see Methods for derivation). This voltage corresponds to the threshold electric field of ~2.6 × 10^6^ V m^−1^ at which corona discharge starts to occur. When corona discharge occurs, the electrode and the filament are bridged together by the corona plasma. The electrons inside the filament would be attracted and neutralized by the positive electrode. The electron density inside the filament decays exponentially when apply the electric field (see the simulation in the Methods). The electric field from the positive charge left behind in the filament cancels the electric field from the positive electrode. Consequently, the effective electric field acting on the filament stays almost constant as the voltage further increases. Meanwhile, the recombination rate between electrons and ions is reduced after corona discharge generation. Thus, the self-heating effect due to recombination in the kHz filaments is minimized and stabilized.

Moreover, similar results were obtained when the direction of laser polarization was changed from the vertical to the horizontal, i.e., the beam pointing stability improvement of the white light laser by the electric field was impregnable to the laser polarization direction. Since the spatial jitter of laser filaments was principally due to the self-induced thermal heating, the higher the filament repetition rate was, the stronger the spatial jitter would be when without the electric field (Fig. 3b). Thus, the extra DC electric field control technique could significantly improve the pointing stability by reducing the beam jitters caused by filament self-heating. This was significantly effective for the repetition rate up to 1 kHz (Fig. 3b).

Since the SC laser was generated through filamentation in air, the stable SC laser should originate from the localization of the filament under the external DC electric field. By imaging the filament cross sectional pattern in the high voltage zone using the technique shown in Fig. 1b, the filament’s local position distribution was recorded by the high speed camera synchronized with the laser pulse. By accumulating 7000 shots (6.1 mJ/30 fs), the SDEV of the filament’s local position as a function of the high voltage is shown in Fig. 3c. The smaller SDEV of the filament’s local position when applying the external field corroborated the improvement of filament pointing stability in air. Moreover, the filament pointing stability varied with the laser repetition rate (see the points at 0 kV in Fig. 3c). The jitter of filament pointing was bigger at higher repetition rate due to filament induced self-heating^19–22^. Applying the extra DC electric field clearly stabilized the filament pointing, especially, at 1 kHz laser repetition rate. This extra DC electric field assisted filament pointing stabilization had much less effect at laser repetition rate at or below 100 Hz. The results were in good agreement with the observation in Figs. 2 and 3a, b. Due to the threshold effect of the DC electric field, the stability of the filaments could not be further improved beyond the threshold for corona generation.

### Spatial position shift

**Fig. 4:**
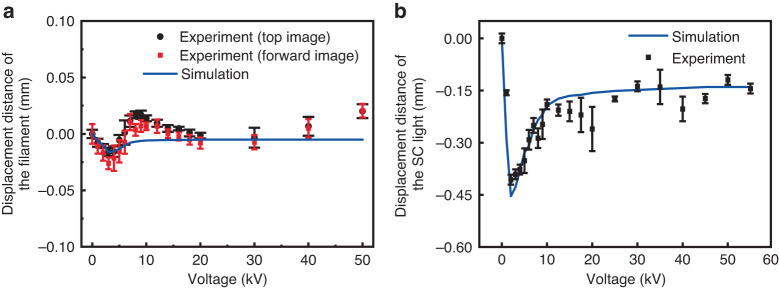
Experimental and numerical results of SC light and filament displacement distance under different voltages. **a** SC light and **b** filament displacement distance in the (horizontal) plane under different applied voltages. The plane contains the electrode and the filament. Position ’0’ represents the initial mean position without the DC electric field. The negative displacement is defined as when the filament is closer to the electrode

The spatial position of the white light laser can be tuned by the external DC electric field. Once a positive DC field was applied on the filament, the center of gravity of the SC light beam pattern ‘suddenly’ moved significantly away from that of the zero field position (Fig. 2f) through Coulomb interaction between the electric field and the filament. Afterwards, when the DC field was further increased, the displacement decreased. As an example, the detailed displacement in the (horizontal) plane containing the electrode and the filament is shown in Fig. 4a (points). The displacement stabilized (see also Fig. 2c–f) when the threshold for corona generation (~2.6 × 10^6 ^V m^−1^)^37^ was reached. Numerical simulation based on the Coulomb interaction is in good agreement with the experimental results (Fig. 4a, solid curve). The details of the simulation is elaborated in the simulation on the filament spatial position shift in the Methods section. The displacements of the filament monitored under different electric fields (The distance between the electrode tip and the filament was 1 mm) are shown in Fig. 4b. The displacement trend of the filament was similar to that of the SC light. The position of the white light changes more markedly than the filament as the voltage increased. The difference is due to the divergence angle of the white light relative to the filament. The white light spot was recorded 1.2 m after the filament (9 cm in length). This means that when the filament moves 1 mm, the white light moves at least 27.7 mm according to the equality ratio of 124.5 cm/4.5 cm. Thus, the position shift of the white light is more significant than that of the filament.

### Spectral stability

**Fig. 5:**
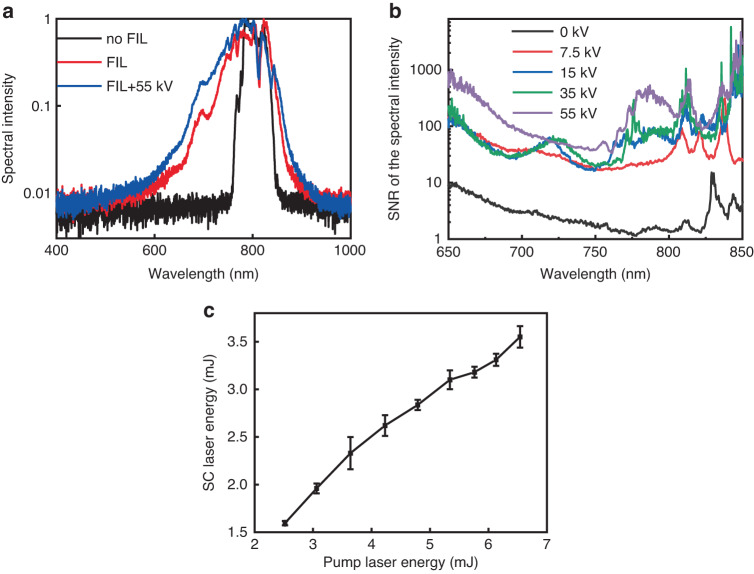
Spectral stability and the energy scaling of the SC laser. **a** The white light spectra after filamentation with (FIL+55kV) and without (FIL) DC electric field. The initial laser spectrum (no FIL) is for comparison. Each spectral distribution was normalized at its maximum. **b** The SNR of the SC spectral intensities under different applied voltages. The laser worked at 1 kHz. **c** The SC laser energy obtained as a function of pump laser energy under 1 m focusing condition

To monitor the intensity fluctuations, an iris was used to select the white light spot from the whole beam pattern. The iris aperture was large enough to let the entire white light spot pass through for spectral intensity measurement (Fig. 1e). Figure 5a shows the spectra of white light laser after filamentation with (FIL + 55 kV) and without (FIL) an external DC electric field. The initial laser spectrum (no FIL) is shown for comparison. The spectra of the FIL and FIL + 55 kV were significantly broadened compared with that of the pump laser pulse which is due to self-phase modulation (SPM) and possibly self-steepening^1,14^. The SC bandwidth was further broadened when the external electric field was applied. Due to the SPM caused by plasma, the frequency change is $\varDelta \omega =\frac{2\pi {e}^{2}}{c{m}_{e}{\omega }_{0}}l\frac{\partial \left[{N}_{e}\left(t\right)\right]}{\partial t}$^14^ with *e* is electron charge, *m*_e_ is electron mass, *N*_e_(*t*) is the electron density and *l* is the effective interaction length of the pump pulse in the plasma. Since ionization in the filament can be enhanced by the external electric field through collision ionization^35^, the plasma density and the effective interaction length would increase leading to broader bandwidth of the SC. Under each electric field condition, 30 spectral intensity curves were captured. The signal to noise ratio (SNR) of the spectral intensity from 650 nm to 850 nm was obtained by the mean value at each wavelength sample divided by the corresponding standard deviation. As the electric field increased, the SNR was significantly improved (Fig. 5b). By using our approach, 3.55 mJ stable SC pulses at 1 kHz was obtained when the input pump pulses (energy/pulse: 6.54 mJ) underwent filamentation directly in air with a 1 m external focusing lens (Fig. 5c).

**Fig. 6:**
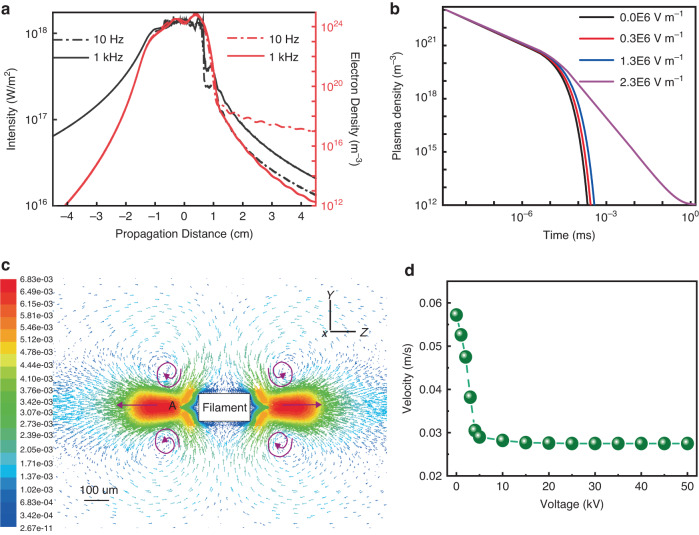
The simulation results of the on-axis plasma density and airflow around the filament. **a** The simulated on-axis laser intensity, plasma density with the laser working at 10 Hz and 1000 Hz. **b** is the simulated time evolution of the plasma density under different electric fields. **c** 2D cross-section of the simulated flow fields around the filament without external electric field. The velocity vectors are colored by velocity magnitude (m s^−1^). The rectangle represents the longitudinal section of the cylindrical filament. The direction of the laser propagation was along the Z axis.The cross-section of the filament was in the XY plane. **d** is the maximum airflow velocity around the filament when applying different voltages. See the details of the simulation in the ’Methods’

### Simulation results

During femtosecond laser filamentation in air, initially there is a plasma density maximum on the optical axis due to laser instantaneous ionization. Typically, the lifetime of the plasma is within a few nanoseconds time scale in an air filament. The thermal self-heating of laser filament comes mainly from the high field ionization and electron-ion recombination. The long thermal decay time (ms) becomes non-negligible at high laser repetition rate. We have carried out a simulation of the interaction.

The filamentation of the high-repetition laser pulse was simulated considering the thermal accumulative effect induced by the self-heat of the filament. Figure 6a shows the laser intensity and plasma density for laser pulses of 10 Hz and 1000 Hz repetition rates, respectively. Affected by the density hole, the intensity increases and the density of plasma decreases for the high-repetition-rate filament^38^. The lifetime of the plasma was also significantly extended (Fig. 6b). The extended lifetime of the plasma was long enough (~ms) to bridge the interval time between two successive pulses. The self-heating effect through plasma recombination would result in air flow near the filament^39^, which gave rise to the jitters of filaments and white light lasers. In order to get an insight into the mechanism of air flow near the laser filaments, we simulated numerically the flow fields in 1 ms after the laser pulse. The 2D velocity vector map of the airflow around the filament is shown in Fig. 6c. The laser pulse propagated along the Z-axis and the cross section of the filament was in the XY plane. The released energy from the plasma recombination heated the ambient air and induced the airflow and vortex around the filament. At both ends of the filament, air vortices emerged and the airflow velocity reached its maximum along the filament propagation path as indicated by the arrows as shown in Fig. 6c. These air disturbances influenced the filament pointing stability. The maximum airflow velocities along the filament propagation axis (e.g., at Point A) by applying different electric fields were calculated as shown in Fig. 6d. Due to the suppression of the plasma recombination, the airflow velocity decreased quickly as the DC field was switched on and further increased. The consequence is that plasma based thermal energy deposition in air was significantly suppressed. Thus, external DC electric field assisted thermal suppression effect contributes to the stability improvement of the laser filament as well as the filament based white light laser in air. Secondly, due to the plasma property of laser filament, external DC electric field provides an extra-control on the spatial localization of laser filament through Coulomb interaction (Fig. 4). All the details of the simulations and the electric field from a coronal discharge are provided in the Methods.

## Discussion

Without the DC electric field, the plasma recombination takes on the order of 1 ns after the laser pulse. After plasma recombination, the excited heavy species de-excite and the stored rotational energy transfers into translational energy (typically takes ∼100 ps), that is the source of heat. The long lifetime (in milliseconds) diffusion of the heat results in the turbulence in air which influences the propagation of the following laser pulse at kHz repetition.

When an extra DC electric field (below the threshold for corona discharge, ~2.6 × 10^6 ^V m^−1^) applied on the filament, the electrons are accelerated and the plasma lifetime are extended which can be up to microseconds dependent on the electric field strength (Fig. 6b). The recombination is restrained and less heat is deposited in the air in the interval time between pulses compared with that without the DC electric field. Consequently, the thermal jitters are restrained. When the DC electric field is larger than the threshold, besides suppressing plasma recombination, the generation of ionic wind from the electrode may also contribute the stabilization process. The ionic wind can overwhelm the wind turbulence by laser filamentation resulting in a steady state. Under the extra electric field, the lifetime of plasma channel as well as the steady state is extended to even millisecond level which is long enough to meet with the next laser pulse (at 1 kHz or higher repetition rate).

Moreover, since the improvement of the stability of the white light laser by the electric field is mainly due to the decrease of the recombination between electrons and ions in the electric field, laser polarization state would not affect the charges’ spatial distribution. Thus, the improvement of pointing stability of the filament is independent of the laser polarization. Furthermore, the displacement of the filament as a whole with respect to the electrode can be explained as follows. Once a small DC field, positive for example, is applied, some electrons inside the filament will be pulled towards the electrode while the positive charges are repelled. The Coulomb attraction between the positive charges and the electrons will keep the filament intact. The filament as a whole will thus be pulled towards the electrode inducing a sudden change of position of the filament. When the electric field is increased, some more electrons will be pulled out of the filament into the electrode. Consequently, the extra positive charges in the filament will be pushed slightly out of the filament. The plasma density inside the filament will be lower. Hence, the attractive force exerted by the electrode is smaller. The filament displacement is thus smaller. Consequently, the higher the electric field is, the smaller the displacement will be (Fig. 4). When the threshold for corona discharge was achieved, the strong plasma at the tip of the electrode will partially shield the field from attracting more electrons into the electrode. Thus, the electric attractive force decreases and the displacement of the filament decreases as the DC electric field increases further. This shielding will stabilize when avalanche ionization (or plasma density) saturates. Further increase of the electric field will only increase the volume of the ionization (plasma) zone. Consequently, the displacement of the filament stabilizes also (Fig. 4).

The displacement of the SC light (laser filament) may result from the guide effect of the plasma waveguide formed by the previous laser pulse under the large DC electric field since the displacement distance cannot be reached in the single laser pulse duration of 30 fs. The minimum plasma density difference to guide a laser beam of spot size ${\omega }_{0}$: $\triangle {N}_{e}^{\min }=\frac{1}{\pi {\omega }_{0}^{2}{r}_{0}}$^40,41^ in which the classical electron radius ${r}_{0}\approx 2.82\times {10}^{-15}m$. For the experimental condition *ω*_0_ = 0.055 mm, $\triangle {N}_{\rm{e}}^{\min }$ should be at least larger than 3.68 × 10^22 ^m^−3^. The plasma density in our case is ~10^23 ^m^−3^, which provides the opportunity to form the plasma waveguide as expected.

In summary, we have experimentally demonstrated stable SC light source generation through femtosecond laser filamentation in air by applying an external DC electric field on the filament. As the electric field increases, the jittering of the beam decreases while the general pointing direction changes and stabilizes at a field higher than about 2.6 × 10^6 ^V m^−1^. This is principally due to the significant suppression of plasma recombination resulting in less thermal heating of the filament zone together with the overwhelmed filament thermal jitter by generating ionic wind from the electrode. Consequently, the beam pointing jitters of a 1 kHz SC light are reduced by more than 2-fold. The stable SC pulse with energy of 3.55 mJ at 1 kHz is achieved by using 6.54 mJ filamenting pump pulse directly in air. Our approach solves the current standing problem of kHz repetition rate filament associated strong jitters. This works at other filamenting wavelengths due to the plasma nature. The findings presented here not only paves a way to generate intense high repetition rate SC light through damage-immune gases and should be beneficial for SC application, but also are crucial and useful for other filament-based applications on secondary radiations, imaging and micromachining of condensed materials etc.

## Materials and methods

### Calculation method of the SDEVs of SC laser and filament scattering angle

The reference axis was the optical axis without filamentation and the high voltage from the starting point of the filament. The calculation methods of the standard deviations (SDEV_*i*_) of each group of SC laser and filament scattering angle were the same. Taking the white light for example, for the *i*^*th*^ group, the length of the reference axis is *l* (the distance between the beginning point of the filament and the screen along the optical axis). The SC laser scattering angle in the *i*^*th*^ group was defined as ${\delta }_{{ij}}=\frac{1}{l}\sqrt{{{x}_{j}}^{2}+{{y}_{j}}^{2}}$ with (*x*_*j*_*, y*_*j*_) being the coordinates of the *j*^th^ white light spot on the screen. The *SDEV*_*i*_ of each group of SC laser scattering angle was calculated to depict the pointing stability where ${{SDEV}}_{i}=\sqrt{\frac{{\sum }_{j=1}^{N}{({\sigma }_{{ij}}-\bar{{{\sigma }_{i}}_{j}})}^{2}}{N-1}}$ with $\bar{{{\sigma }_{i}}_{j}}=\frac{1}{N}{\sum }_{j=1}^{N}{\sigma }_{{ij}}$ being the average of the group scattering angles of the white light and *N* = 300.

### Simulation on high-repetition rate laser filamentation

Pulse cumulative effect leads to a “low density hole” along high-repetition laser filament^19^. The NLSE corrected by the “low density hole” for describing the laser pulse propagation under different repetition rates can be expressed as^42,43^:

1 $$\frac{\partial E}{\partial z}=i\frac{1}{2{k}_{0}}{\triangle }_{\perp }E-i\frac{{k}^{{\rm{\text{'}\text{'}}}}}{2}\frac{{\partial }^{2}E}{\partial {t}^{2}}+i\frac{{\omega }_{0}}{c}{n}_{2}{IE}-\frac{{\beta }^{K}}{2}{I}^{K-1}E-\frac{\sigma }{2}\left(1+i{\omega }_{0}\tau \right)\rho E+i{k}_{0}\triangle {nE}$$

2 $$\frac{\partial \rho }{\partial t}=\frac{{\beta }^{K}}{{K{\hslash}\omega }_{0}}\left(1-\frac{\rho }{{\rho }_{{air}}}\right){I}^{K}$$

The values of the parameters in the propagation model (Eqs. (1) and (2)) vary with the air density and are described detail in our recent work^38^.

The filament heating process can be regarded as an isochoric (constant volume) process, the plasma recombination led to the peak temperature variation of air $\triangle {T}_{{peak}}$ can be calculated according to ref. ^38^.

The energy deposition in the air can be described as^44^:

3 $$\frac{\partial T}{\partial t}=\alpha \left[\frac{1}{r}\frac{\partial }{\partial r}\left(r\frac{\partial T}{\partial r}\right)+\frac{{\partial }^{2}T}{\partial {z}^{2}}\right]$$

4 $${\rho }_{{index}}=\frac{{\rho }_{{air}}}{{\rho }_{{at}}}=\frac{T(0)}{T(t)}$$

where $\alpha =\frac{\chi }{{c}_{p}}$ is thermal diffusivity, $\chi =24.6\times {10}^{-3}{\rm{W}}/({\rm{m}}\,\cdot\, {\rm{K}})$ is thermal conductivity of air. The process of the energy deposition can be regarded as isobaric process since the air pressure remains constant, so ${c}_{p}=7{k}_{{\rm{B}}}{\rho }_{{at}}/2$ is heat capacities at constant pressure^44^.

The “air density hole” can lead to the refractive index change:

5 $$\triangle n=({n}_{0}-1)\frac{T\left(t\right)-T(0)}{T(0)}$$

where the refractive index of the ambient air ${n}_{0}=1.000275$.

The envelope of the electric field ε of the input laser beam could be written as^45^:

6 $$\varepsilon \left(r,t,z=0\right)={\varepsilon }_{0}\exp (-{r}^{2}/{w}_{0}^{2}-{t}^{2}/{t}_{p}^{2}-i{k}_{0}{r}^{2}/2f)$$

where ${w}_{0}={w}_{f}{\left(1+{d}^{2}/{z}_{f}^{2}\right)}^{1/2}$ was transverse beam waist at the distance d before the geometrical focus, the initial beam waist was ${w}_{f}=4.4\,{\rm{mm}}$, the laser pulse duration was ${t}_{p}=30\,{\rm{fs}}$, the Rayleigh length was ${z}_{f}=\pi {w}_{f}^{2}{n}_{0}/{\lambda }_{0}$, the lens focal length was $f=50\,{\rm{cm}}$, and the curvature of the laser pulse at the distance d from the linear focus was $f=d+{z}_{f}^{2}/d$.

### Simulation of the static electric field from a corona discharge electrode

There are two stages in the simulation: the first stage is when the electric field is below 2.6 × 10^6 ^V m^−1^ (the threshold of generating the corona discharge around the electrode tip); the second stage is when the electric field is beyond 2.6 × 10^6 ^V m^−1^ at which the electrode tip and the filament are connected by a short plasma.

**Fig. 7:**
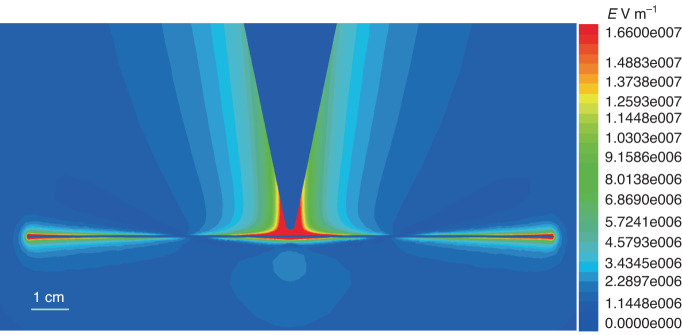
Static electric field distribution in the horizontal plane with the filament plasma channel. The applied high voltage was 4 kV

For the first stage, the effective electric field *E*_eff_ was equivalent to the electric filed created by the voltage applied on the electrode (*E*_v_) and was calculated based on the Poisson Eq. (7) and Maxwell Eq. (8).

7 $$\nabla \,\cdot\,\left({\varepsilon }_{{\rm{r}}} \,\cdot\, {\varepsilon }_{0}\nabla \varPhi \right)=-{\rho }_{{\rm{V}}}$$

8 $$E=-\nabla \varPhi$$

where ${\varepsilon }_{r}$ is the relatively dielectric constant, ${\varepsilon }_{0}$ is the permittivity of vacuum, *Φ* is the electric scalar potential, and ${\rho }_{{\rm{V}}}$ is the density of volume charges. The distribution of DC electric field can be solved by Eqs. (7) and (8). These equations are solved by a computer in 3D based on finite element analysis which is widely used in electromagnetic field simulation. The calculation model was established, at one-to-one scale, according to the condition in our experiment. The filament plasma channel was considered as a conductor with the bulk conductivity of 100 siemens m^−1^^46^. The simulation result is shown in Fig. 7. The voltage is 4 kV. The maximum electric field along the filament surface was taken as the effective electric field applied on the filament.

For the second stage, the filament and the electrode tip were connected and the electrons inside the filament would move to the electrode tip. The plasma shielding effect should be taken into consideration.

The electrode and the filament will be connected by the corona discharge plasma with the density of 10^14^cm^−3^^47^ which can be equivalent to a conductor. The electrons inside the filament are then forced by the electric field to move to the electrode tip along the ‘conductor’.

The electron mobility is^48^:

9 $${\mu }_{{\rm{e}}}\left({m}^{2}/V\,\cdot\, s\right)=-\frac{{N}_{0}}{3N}{\left(\frac{5\times {10}^{5}+{E}_{0}}{1.9\times {10}^{4}+26.7\times {E}_{0}}\right)}^{0.6}$$

10 $${E}_{0}={{E}_{{\rm{v}}}N}_{0}/N$$

where *E* is the electric field, *N* and *N*_*0*_ are the molecule densities in the considered condition and in normal conditions, respectively.

In the electric field, the resistance of the plasma conductor over the pathway *d* expresses as^49^:

11 $$R(E)=\frac{1}{e{u}_{{\rm{e}}}A}{\int }_{0}^{d}\frac{d\overrightarrow{r}}{\rho (\overrightarrow{r})}$$

where *A* is the cross-section of the conducting area and ${\rho }_{{\rm{e}}}$ is the free electron density. To simplify the simulation, the cross-section *A* of plasma conductor is assumed to be the area of 100 ${\rm{\mu }}{\rm{m}}$ diameter same with the filament diameter and $\rho =1{0}^{14}\text{c}{\text{m}}^{-3}$^46^. As a result, Eq. (11) is simplified as:

12 $$R(E)=\frac{d}{e{u}_{{\rm{e}}}A\rho }$$

Before the electric field reaching 2.6 × 10^6 ^V m^−1^, the electrode and the filament form a capacitor, and its capacitance can be calculated by:

13 $$C={Q}_{{\rm{e}}(0)}/U$$

where ${Q}_{{\rm{e}}(0)}=V* {n}_{{\rm{e}}}$ is the total induced charges of the filament. *V* is the volume of the filament and ${n}_{e}$ is the electron density inside the filament. *U* = 2.6 × 10^6 ^× *d* is the voltage difference between the electrode and the filament. *d* is the distance from the electrode tip to the filament.

In analogy with the capacitor discharging process, the reduction of the induced negative charges of the filament ${Q}_{{\rm{e}}(t)}$ can be described as:

14 $${Q}_{{\rm{e}}(t)} \sim {Q}_{{\rm{e}}(0)}\exp \left(-\frac{t}{{CR}(E)}\right)$$

Then, the filament is positively charged and its charge ${Q}_{i(t)}$ is equal to the reduction of the negative charge ${Q}_{{\rm{e}}(t)}$. The electric field (${E}_{{\rm{eff}}}$) near the filament created by the electrode would be cancelled by the positive ions (${E}_{{\rm{ion}}}$) which can be simply written as:

15 $${E}_{{\rm{ion}}} \sim \frac{{Q}_{i\left(t\right)}}{4* \pi * {\varepsilon }_{0}* {R}^{2}}$$

16 $${{E}_{{\rm{eff}}}=E}_{{\rm{v}}}-{E}_{{\rm{ion}}}$$

where the ${\varepsilon }_{0}$ is the permittivity of vacuum and the *R* is the diameter of the filament.

### Simulation on plasma decay time in the filament

Under an external DC electric field, dominant processes involved in the wake of plasma channel induced by femtosecond laser pulse include electron-ion recombination, ion-ion recombination, impact ionization, attachments and dissociative attachments of electrons to oxygen molecules. Therefore, time evolutions of electron density *n*_e_, positive ion density *n*_p_, and negative ion density *n*_n_ can be described by following equations^48^:

17 $$\left\{\begin{array}{c}\frac{\partial {n}_{{\rm{e}}}}{\partial t}=\gamma {n}_{{\rm{e}}}-\eta {n}_{{\rm{e}}}-{\beta }_{{\rm{ep}}}{n}_{{\rm{e}}}{n}_{{\rm{p}}}\\ \frac{\partial {n}_{{\rm{p}}}}{\partial t}=\gamma {n}_{{\rm{e}}}-{\beta }_{{\rm{ep}}}{n}_{{\rm{e}}}{n}_{{\rm{p}}}-{\beta }_{{\rm{np}}}{n}_{{\rm{n}}}{n}_{{\rm{p}}}\\ \frac{\partial {n}_{{\rm{n}}}}{\partial t}=\eta {n}_{{\rm{e}}}-{\beta }_{{\rm{np}}}{n}_{{\rm{n}}}{n}_{{\rm{p}}}\end{array}\right.$$

where impact ionization coefficient was $\gamma =\left(\frac{N}{{N}_{0}}\right)\frac{5.7\times {10}^{9}{\alpha }^{5}}{1+0.3{\alpha }^{2.5}}$^48^. *N* was air density, *N*_0_ = 2.699$\times$10^25 ^m^−3^, $\alpha =3.34\times {10}^{-7}E\frac{N}{{N}_{0}}$, and *E* was strength of external DC electric field. Attachment coefficient of electron to O_2_ was $\eta =2.5\times {10}^{7}{\mathrm{s}}^{-1}$. The coefficients of electron-ion recombination and ion-ion recombination were ${\beta }_{{\rm{ep}}}=2.2\times {10}^{-13}{\mathrm{m}}^{3}/{\mathrm{s}}$ and ${\beta }_{{\rm{np}}}=2.2\times {10}^{-13}{\mathrm{m}}^{3}{\mathrm{/s}}$, respectively^48^.

### Simulation on filament spatial position shift

**Fig. 8:**
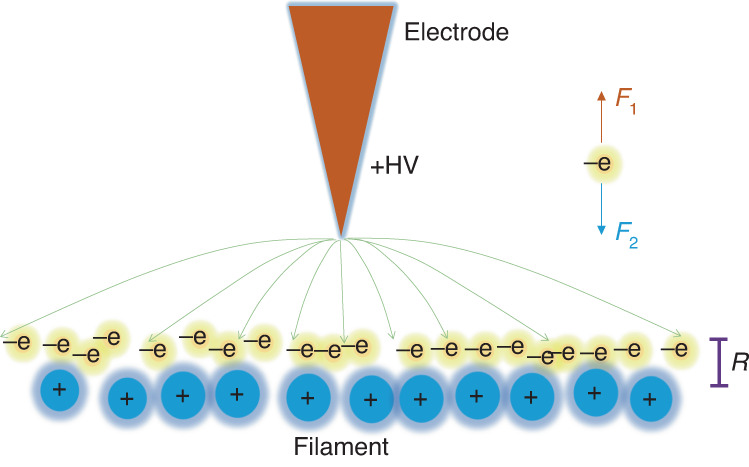
Schematic diagram of the high voltage electrode controlled filament via the electric field lines as if it were a fan. R is the diameter of the filament; F_1_ and F_2_ are the attractive electric forces in the opposite direction induced by the positive high-voltage electric field and the positive ions, respectively

In the positive static electric field, the light electrons inside the filament tend to move toward the positive electrode, while the heavy positive ions are pushed slightly away leading to the charge separation. The electron then is attracted simultaneously by the positive static high-voltage electric field and positive ions in the opposite directions, as shown inset in Fig. 8.

According to our experimental observation, the filament diameter stayed nearly constant as the applied voltage increases. Then the filament diameter was assumed to be 100 ${\rm{\mu }}{\rm{m}}$.

Base on the Eqs. (9–11), the displacement (*S*) of the electrons in the plasma lifetime (*t*) is approximated as:

18 $$S \sim {\mu }_{{\rm{e}}}* {E}_{{\rm{eff}}}* t$$

### Simulation on gas flow field around filament

The filament induced heat flux source was set by user-defined functions (UDF) programmed with C language, which was then interpreted and complied by Fluent 6.3. In the experiments, when the pump laser with 6.12 mJ was focused to generate the air filaments with a diameter of 100 μm and a length of 9 cm, the total deposited energy of the filament (pump laser energy subtracts laser energy after filament) was 1.24 mJ. In the 2D simulation, a uniform energy density inside the filament as in the experiment is under consideration. To reduce the calculation time, the length and diameter of the filament were set as 200 μm and 100 μm, respectively. Besides, the filament was treated as a cylinder in 3D. Then the total deposited energy was estimated to be 2.75 × 10^−3^ mJ which was the energy to initiate the airflow disturbances. The heating time was assumed to be the same as the plasma lifetime, after which the deposited energy began to thermally diffuse outward. When applying different DC electric fields on the filament, the plasma lifetime was extended and proportional to the DC electric field. As a consequence, plasma recombination based thermal energy deposition in air was significantly suppressed and therefore calculated. The deposited laser energy under the DC electric field was then used to calculate the airflow velocity under the condition.

**Fig. 9:**
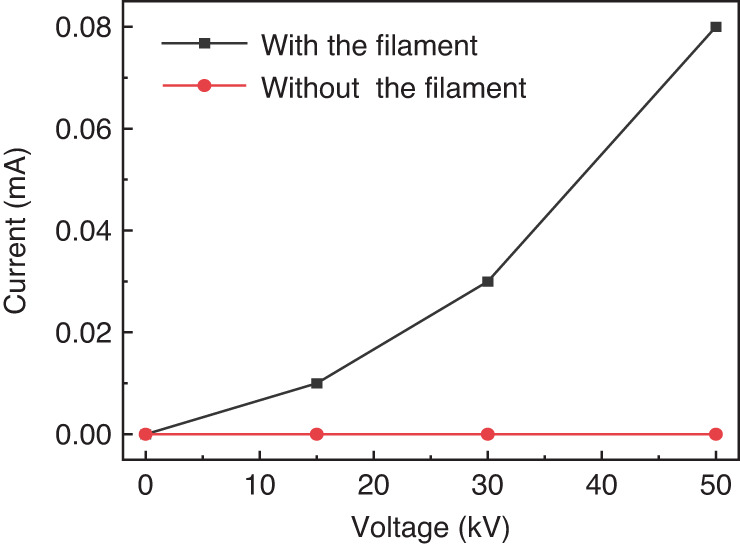
The output current as a function of the applied voltages

### Time averaged current at the electric field producing electrodes

The output current as a function of the applied high voltage on the electrodes was measured as shown in Fig. 9. The high voltage power supply is a DC constant voltage source. Without the filament, the output current of coronal discharges from the electrodes stays almost at 0 mA. The current clearly increases with the increase of the applied voltage when the filament was created around the electric field producing electrode.
